# Association between soybean product consumption and executive function in Chinese Tibetan children and adolescents

**DOI:** 10.3389/fnut.2024.1348918

**Published:** 2024-02-29

**Authors:** Xiaojian Yin, Feng Zhang, Cunjian Bi, Yuan Liu, Yaru Guo, Pengwei Sun, Jun Hong

**Affiliations:** ^1^Department of Physical Education, Shanghai Institute of Technology, Shanghai, China; ^2^Key Laboratory of Adolescent Health Assessment and Exercise Intervention of Ministry of Education, East China Normal University, Shanghai, China; ^3^College of Physical Education and Health, East China Normal University, Shanghai, China; ^4^Sports Health Promotion Center, Chizhou University, Chizhou, China; ^5^Physical Education College of Shanghai University, Shanghai, China

**Keywords:** association, soybean product consumption, executive function, Chinese Tibetan, children and adolescents

## Abstract

**Objective:**

This sought to explore the association between soybean product consumption and executive function (EF) in Chinese Tibetan adolescents in high-altitude areas.

**Methods:**

A total of 1,184 Tibetan children and adolescents were tested on demographic variables, soybean product consumption, and executive function in Lhasa and Nagchu regions of Tibet, China, using stratified whole population sampling. One-way ANOVA, linear regression analysis, and logistic regression analysis were used to analyze the associations existing between soybean product consumption and executive function.

**Results:**

The proportions of Hardly ever, Occasionally, and Often in Soy Consumption among Tibetan children and adolescents in high altitude areas of Tibet, China were 21.7, 50.3, and 28.0%, respectively. The difference in 2 back reaction time among Tibetan children and adolescents with different soybean product consumption was statistically significant (*F* = 6.374, *p* = 0.002). The difference in conversion function reaction time was also statistically significant (*F* = 8.129, *p* < 0.001). Taking the soybean product consumption ≥6 t/w group as the reference group, after adjusting the relevant factors, those with soybean product consumption ≤1 t/w showed a statistically significant increase in Inhibit Function Dysfunction (OR = 1.844, 95% CI: 1.152, 2.951) and Conversion Function Dysfunction (OR = 2.008, 95% CI: 1.106, 3.646) had an increased risk of Conversion Function Dysfunction (OR = 2.008, 95% CI: 1.106, 3.646), which was significantly different (*p* < 0.05).

**Conclusion:**

There is an association between soybean product consumption and inhibitory control and translational flexibility of brain executive functions in Chinese Tibetan children and children and adolescents at high altitude.

## 1 Introduction

Executive functions are critical to child and adolescent development, enabling children and children and adolescents to effectively focus their attention, multitask, understand and manage their emotions, and enhance their social skills (1–3). Executive functions are highly correlated with child and adolescent development and are important predictors of behavioral functioning, academic achievement, and even other outcomes such as athletic achievement, academic performance, mental health, social well-being, health, wealth, and quality of life (4–6). It is now widely recognized in the academic community that there are three core functions of executive function: inhibitory, working memory, and translational. On top of the three core executive functions, the brain builds higher-order executive functions, such as logical reasoning, problem solving, and task planning, with the three subcomponents being both independent and interrelated (7). Executive functions are essential skills for adolescents’ healthy physical and mental development, success in school and life, and cognitive, social, and psychological development (8–19). On the contrary, when children and adolescents have executive function deficits, such as attention deficit hyperactivity disorder (ADHD) and aggressive behaviors, it can cause heavy psychological and physical stress on the family (20, 21). Thus, executive function plays an important role in the healthy development of children and adolescents.

Soy products play an important role in people’s daily diet and bring many health benefits (22). The so-called soy products usually include various kinds of food made from soybeans and mung beans as the main raw materials, such as edamame, fermented bean curd, soybean paste, soy milk, dried beans and other foods. Soy products are rich in high-quality protein and high in unsaturated fatty acids. Research has found that soybeans and their products have a high nutritional value, protein content of about 35%, and is a high-quality protein, containing a variety of essential amino acids needed by the human body (23). The Dietary Guidelines for Chinese Residents (2022) recommends a weekly intake of 105–175 g of soybeans or an equivalent amount of soybean products, while the daily intake of soybeans and soybean products by Chinese residents is only 10.3 g, which is far below the recommended intake (24). This shows that research on soybean products should be emphasized in the future in order to better increase the consumption of soybean products.

Previous studies on executive function in children and adolescents have focused on physical activity and executive function, sugary drinks and executive function, and relatively few studies on soybean product consumption and executive function in dietary behavior (25, 26). In addition, no studies have been found on soybean product consumption and executive function in Tibetan children and adolescents at high altitude (27). Tibetan children and adolescents living in Tibet, China, live at high altitude all year round, which has certain effects on brain health and plateau adaptation (28, 29). This reason, it is necessary to conduct research on the brain executive function and soybean product consumption of their Tibetan children and adolescents, in order to promote the healthy development of the brain.

## 2 Methods

### 2.1 Participants

**Figure 1:**
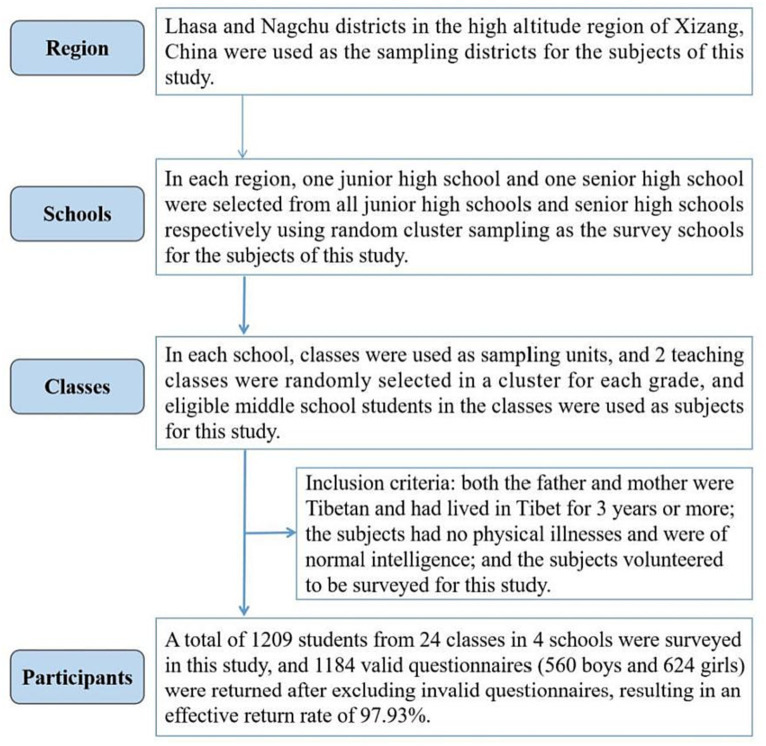
Sampling flow of Chinese Tibetan children and adolescents subjects in high altitude.

This study used three stages of stratified whole cluster sampling method for the sampling of subjects. In the first stage, the regions of Lhasa (3,650 meters) and Nagchu (4,450 meters) in the high altitude region of Tibet, China were used as the sampling regions for the subjects of this study. In the second stage, one junior high school and one senior high school in each region among all junior high schools and senior high schools were selected as the survey schools for the subjects of this study by using random whole cluster sampling method. In the third stage, in each school, 2 instructional classes were randomly selected in a whole cluster for each grade using classes as the sampling unit, and the eligible middle school students in the classes served as the subjects of this study. The inclusion criteria for this study were: both the father and mother were Tibetan and had lived in Tibet, China for 3 years or more; the subjects had no physical illnesses and were of normal intelligence; and the subjects volunteered to be surveyed for this study. In the end, a total of 1,209 people from 24 classes in 4 schools were surveyed, and after 25 invalid questionnaires were excluded, 1,184 valid questionnaires were returned (560 boys and 624 girls), with an effective return rate of 97.93%. The mean age of the participants was (15.78 ± 1.68) years. The survey period for this study was April–November 2020. The specific sampling process is shown in Figure 1.

Written informed consent for this study survey was obtained from the students’ parents and themselves, and the survey was conducted after signing a written informed consent form. This study was approved by the Human Ethics Committee of East China Normal University (No.: HR0782020).

### 2.2 Basic demographic information

The survey of basic demographic information in this study included the information of the subjects’ area, school, grade, class, gender, year of birth, and test date. The subjects were asked to fill in the basic information truthfully according to their actual situation. The age of the subjects was calculated according to their birth date and test date, and the age of the subjects was calculated according to the whole year if it was less than a whole year.

### 2.3 Soy consumption

The soy products investigated in this study mainly included the frequency of soy products consumed by the subjects in the past week. The standard of soy product consumption is about 200 g for a standard cup of soy milk, 200 g for a standard bowl of tofu brain, and 200 g for a serving of soy product. The respondents were asked to recall the frequency of consumption of soybean products in the past 7 days according to their own situation and fill in the questionnaire (24, 30).

### 2.4 Executive function

The test of executive function in this study includes subjects’ inhibitory function, refreshing function, and switching function. (1) Inhibitory function refers to a person’s ability to control his or her attention, behavior, thoughts, and emotions in order to override strong internal tendencies or external temptations in order to perform more appropriate or needed tasks. (2) Refreshing function, also known as working memory function, refers to the ability to form deep memories of external information and process the information in a very short period of time. The ability to temporarily hold relevant information in a very short period of time in the relevant specific areas of the brain where the individual can quickly extract it or carry out a step of processing to deal with such information. (3) Switching function refers to the ability to switch smoothly between different cognitive tasks, also known as cognitive flexibility. Switching function helps individuals quickly adjust their cognitive and behavioral styles to adapt to environmental changes.

The inhibitory function in this study was tested using Flanker’s experimental paradigm for the subjects. The refreshing function was tested using 1-back and 2-back experimental paradigms on the subjects. The switching function was tested on subjects using the More-odd Shifting experimental paradigm. The testing of the execution function required the subjects to be tested in the computer room of the school, and the testing program was based on the E-prime 1.1 software system. Participants were asked to react quickly to the letters and colors that appeared on the computer screen and to press the appropriate keyboard keys while ensuring that they were correct. The specific test methods were referred to the specific test methods described in the previously published literature (31).

### 2.5 Covariates

The covariates tested included father’s education, mother’s education, sleep adequacy, waist circumference, moderate to high intensity physical activity, and maximum oxygen uptake in addition to basic demographic information. Father’s education and mother’s education were categorized as Primary School or Below, secondary and high school, Junior college or above, and sleep adequacy was categorized as Adequate (≥8 h/d) and Insufficient(<8 h/d) (32). Waist circumference was measured according to the testing method and instrument required by the China National Survey on Students’ Physical Fitness and Health, and the results were accurate to 0.1 cm (33). MVPA was calculated according to the time of the items of medium-intensity and high-intensity physical activity in the questionnaire of the China National Survey on Students’ Physical Fitness and Health, and the subjects were mainly tested for the time and frequency of MVPA per day in the past 7 days, so as to calculate the average MVPA per day of the subjects in the past 7 days. VO_2max_ was calculated by using the 20-meter round-trip running test and converted to VO_2max_ by the formula. 20-meter round-trip running was tested according to Léger’s test method (34). The test method is that the tester runs back and forth between two lines at a distance of 20 m, and each completion of 20 m is recorded as 1 lap (times), the running speed is controlled by the music, the initial speed is 8.0 km/h at the beginning, the speed is 9.0 km/h at the second minute, and the speed is accelerated by one speed level every minute, i.e., each time the speed is increased by 0.5 km/h. The tester tries to complete the speed level as much as possible, and the tester can not follow the rhythm to reach the 20 m end of the test for 2 times consecutively. The test is completed when the 20 m endline cannot be reached at tempo for 2 consecutive times, and the speed of the last stage is recorded to obtain the maximal aerobic speed (MAS). The complete 20 m SRT consists of 247 laps in 21 levels, and the maximum aerobic speed is 18.5 km/h. The VO_2max_ level of children and adolescents aged 8–19 years was mainly projected based on the developed equation, which is as follows: VO_2max_ = 31.025 + 3.238 × S − 3.248 × age + 0.1536 × S × age. × age. s denotes the tester’s final maximal speed, and S = 8 + 0.5 × the highest level reached by the tester (35).

### 2.6 Quality control

The questionnaire survey of the subjects was carried out by professional investigators who were qualified after training and examination. The problems of the subjects in the process of filling out the questionnaire were explained and answered in a timely manner. The questionnaire filling process requires subjects to fill out the questionnaire independently without time constraints, according to their own actual situation. The questionnaire is required to fill in and retrieve the questionnaire on the spot. The returned questionnaires were briefly checked, and the subjects were asked to complete the questionnaires in a timely manner if there were omissions or wrong entries, in order to reduce the number of invalid questionnaires and guarantee the quality of the survey.

### 2.7 Statistical analysis

The subjects in this study were categorized according to age and gender. Continuous variables were expressed in terms of mean and standard deviation, and categorical variables were expressed in terms of percentage. The frequency of Soy Consumption was classified as hardly ≤1 time/week, occasionally 2–5 time/week, and often ≥6 time/week. Each calculation was based on 1 standard cup of soymilk of about 200 g; 1 standard bowl of tofu brain of about 200 g; 1 serving of soy products of about 200 g; and 1 standard bowl of tofu brain of about 200 g. Each calculation is based on 1 standard cup of soymilk of about 200 g; 1 standard bowl of tofu brain of about 200 g; and 1 serving of soya products of 200 g. The executive function was expressed as mean and standard deviation. Comparisons of executive function between subjects with different Soy Consumption were made by one-way ANOVA, and the relationship between Soy Consumption and executive function was analyzed by linear regression analysis. The executive function reaction time higher than 1 standard deviation was also used as the cut-off value to define the presence of executive dysfunction, which was categorized into two groups. The analysis was performed by means of binary logistic regression analysis. Model 1 controlled for age, gender, father’s education, and mother’s education. Model 2 controlled for factors such as waist circumference, moderate to high intensity physical activity per day, and maximal oxygen uptake (VO_2max_) on the basis of model 1. Data were analyzed using IBM SPSS 25.0 software (IBM Corp., Armonk, NY, United States) with a test level of *a* = 0.05.

## 3 Results

In this study, soybean product consumption and executive function were tested and investigated in 1184 (560 boys, 47.3%) Tibetan children and adolescents in the high altitude area of Tibet, China, and the mean age of the investigators was (15.78 ± 1.68) years. The proportion of Father’s education in primary school or below, secondary and high school, Junior college or above was 63.6, 24.4 and 12.0%, respectively, the difference was statistically significant (χ^2^ = 15.039, *p* = 0.005). The proportion of mothers whose education was primary school or below, secondary and high school, and Junior college or above was 72.6, 15.3, and 12.1%, respectively, the difference was statistically significant (χ^2^ = 19.288, *p* < 0.01). The mean circumference was (68.56 ± 7.21) cm. The results of this study showed that the percentages of Hardly ever, Occasionally, and Often in Soy Consumption among Tibetan children and adolescents in the high altitude area of Tibet, China were 21.7, 50.3, and 28.0%, respectively, and the difference was not statistically significant in comparison (χ^2^ = 2.029, *p* = 0.363). The results of this study show that there is a difference in Soy Consumption among Tibetan children and adolescents in terms of Father’s education and Mother’s education, and the difference is statistically significant when compared (*χ*^2^ = 15.039, 19.288, *p* < 0.01) (see Table 1).

**Table 1:** Basic status of soybean product consumption among Chinese Tibetan children and adolescents.

Classifications	Soy consumption			Total	*χ*^2^-value/*F*-value	*p*-values
	Hardly ever	Occasionally	Often			
N	257 (21.7)	595 (50.3)	332 (28.0)	1,184	2.029	0.363
Age(years)	15.53 ± 1.61	15.8 ± 1.69	15.96 ± 1.69	15.78 ± 1.68	4.896	0.008
Sex						
Boys	131 (51.0)	279 (46.9)	150 (45.2)	560 (47.3)	2.029	0.363
Girls	126 (49.0)	316 (53.1)	182 (54.8)	624 (52.7)		
Father’s education						
Primary school or below	119 (53.6)	340 (64.4)	201 (70)	660 (63.6)	15.039	0.005
Secondary and high school	68 (30.6)	128 (24.2)	57 (19.9)	253 (24.4)		
Junior college or above	35 (15.8)	60 (11.4)	29 (10.1)	124 (12.0)		
Mother’s education						
Primary school or below	142 (64.8)	386 (72.8)	222 (78.2)	750 (72.6)	19.288	0.001
Secondary and high school	51 (23.3)	81 (15.3)	26 (9.2)	158 (15.3)		
Junior college or above	26 (11.9)	63 (11.9)	36 (12.7)	125 (12.1)		
Adequate sleep						
Adequate	123 (48.2)	244 (41.4)	150 (45.6)	517 (44.1)	3.775	0.151
Insufficient	132 (51.8)	345 (58.6)	179 (54.4)	656 (55.9)		
Waist circumference	69.33 ± 8.42	68.40 ± 6.74	68.25 ± 6.98	68.56 ± 7.21	1.929	0.146
MVPA	42.02 ± 22.97	42.20 ± 24.05	40.58 ± 21.47	41.71 ± 23.11	0.554	0.575
VO_2max_	36.75 ± 6.24	37.13 ± 5.15	37.46 ± 5.75	37.14 ± 5.57	1.156	0.315

Soy Consumption, Hardly ever ≤ 1 time/week, Occasionally 2–5 time/week, Often ≥ 6 time/week. Each calculation uses 1 standard cup of soy milk about 200 g; 1 Standard bowl of bean curd about 200 g calculation; One serving of soy products is calculated as 200 g. Descriptive statistics are presented as mean (standard deviation) and number (percentage) for continuous and categorical.

Each calculation uses 1 standard cup of soy milk about 200 g; 1 Standard bowl of bean curd about 200 g calculation; One serving of soy products is calculated as 200 g. Descriptive statistics are presented as mean (standard deviation) and number (percentage) for continuous and categorical.

The results of this study showed that the difference in 2 back Reaction Time between different soybean product consumption Tibetan children and adolescents in high altitude areas of Tibet, China, was statistically significant when compared (*F* = 6.374, *p* = 0.002). Conversion function reaction time was also statistically significant when compared. The difference was also statistically significant (*F* = 8.129, *p* < 0.001). In terms of the subfunctions of the executive function, Consistent reaction time, Inconsistent reaction time, Switching reaction time were worse compared to each other, and the differences were also statistically significant (*F* = 5.293, 5.563, 8.536, *p* < 0.05) (see Table 2).

**Table 2:** One-way analysis of executive function status of Tibetan children and adolescents with different soybean product consumption.

Executive function	Soy consumption	*N*	M	SD	*F-*value	*p*-values
Consistent reaction time	≤1 t/w	257	790.63	97.40	5.293	0.005
	2–5 t/w	595	775.91	83.09		
	≥6 t/w	332	767.68	79.44		
Inconsistent reaction time	≤1 t/w	257	811.16	97.40	5.563	0.004
	2–5 t/w	595	796.59	83.23		
	≥6 t/w	332	787.55	79.28		
Inhibit function reaction time	≤1 t/w	257	20.53	5.92	1.954	0.142
	2–5 t/w	595	20.69	6.17		
	≥6 t/w	332	19.87	6.17		
1back reaction time	≤1 t/w	257	997.74	314.59	1.404	0.246
	2–5 t/w	595	975.69	310.37		
	≥6 t/w	332	954.26	317.61		
2back reaction time	≤1 t/w	257	1178.82	319.45	6.374	0.002
	2–5 t/w	595	1091.11	369.70		
	≥6 t/w	332	1086.20	357.80		
Switching reaction time	≤1 t/w	257	1178.99	265.26	8.536	<0.001
	2–5 t/w	595	1135.23	247.70		
	≥6 t/w	332	1094.04	234.53		
Size parity reaction time	≤1 t/w	257	758.48	114.80	0.940	0.391
	2–5 t/w	595	751.74	113.43		
	≥6 t/w	332	745.21	124.38		
Conversion function reaction time	≤1 t/w	257	420.51	235.11	8.129	<0.001
	2–5 t/w	595	383.49	218.32		
	≥6 t/w	332	348.83	189.22		

Soy Consumption, Hardly ever ≤ 1 time/week, Occasionally 2–5 time/week, Often ≥ 6 time/week. Each calculation uses 1 standard cup of soy milk about 200 g; 1 Standard bowl of bean curd about 200 g calculation; One serving of soy products is calculated as 200 g. Descriptive statistics are presented as mean (standard deviation) for continuous and categorical.N, number; M, mean; SD, standard deviation.

Linear regression analysis was performed with Executive Function reaction time as the dependent variable and soybean product consumption as the independent variable among Tibetan children and adolescents at high altitude in Tibet, China. Model 1 was a crude model, model 2 controlled for age, gender, father’s education, mother’s education, etc., and model 3 controlled for waist circumference, daily moderate-high-intensity physical activity, and VO_2max_ on the basis of model 2. The results showed that after adjusting for the relevant factors and using the soybean product consumption ≥6 t/w group as the reference group, the reaction time of those with soybean product consumption ≤1 t/w was analyzed by linear regression. Consistent reaction time and Consistent reaction time were significantly higher in those with soybean product consumption ≤1 t/w, with 16.666 ms and 16.458 ms, respectively, and the differences were statistically significant (*p <* 0.05). In addition, with the soybean product consumption ≥6 t/w group as the reference group, after adjusting the relevant factors, the Switching reaction time and Conversion function reaction time of those with soybean product consumption ≤1 t/w increased by 43.666 ms and 16.458 ms, respectively, and the difference was statistically significant (*p*<0.05). After adjusting the relevant factors, the Switching reaction time and Conversion function reaction time of those with soybean product consumption ≤1 t/w increased by 43.765 ms and 56.485 ms, respectively, and the differences were statistically significant (*p* < 0.05). Overall, with the decrease of soybean product consumption, the Conversion function reaction time showed an increasing trend (see Table 3).

**Table 3:** Linear regression analysis of soybean product consumption and executive function in Tibetan children and adolescents at high altitude.

Executive function	Est (95% CI)		
	Model 1	Model 2	Model 3
Consistent reaction time			
≥6 t/w	0.000	0.000	0.000
2–5 t/w	17.656 (5.075, 30.237)^b^	17.622 (6.307, 28.937)^b^	11.005 (0.555, 21.455)^a^
≤1 t/w	30.871 (15.442, 46.300)^c^	29.795 (15.765, 43.825)^c^	16.666 (3.647, 29.686)^a^
*p* for trend	<0.001	<0.001	<0.001
Consistent reaction time			
≥6 t/w	0.000	0.000	0.000
2–5 t/w	18.056 (5.448, 30.664)^b^	17.915 (6.624, 29.206)^b^	11.316 (0.924, 21.707)^a^
≤1 t/w	31.131 (15.668, 46.593)^c^	29.651 (15.651, 43.651)^c^	16.458 (3.510, 29.405)^a^
*p* for trend	<0.001	<0.001	<0.001
Inhibit function reaction time			
≥6 t/w	0.000	0.000	0.000
2–5 t/w	0.400 (−0.517, 1.316)	0.293 (−0.606, 1.192)	0.310 (−0.578, 1.198)
≤1 t/w	0.260 (−0.864, 1.384)	−0.144 (−1.259, 0.970)	−0.208 (−1.314, 0.898)
*p* for trend	0.693	<0.001	<0.001
1back reaction time			
≥6 t/w	0.000	0.000	0.000
2–5 t/w	30.315 (−16.454, 77.085)	29.608 (−16.070, 75.287)	8.456 (−35.319, 52.231)
≤1 t/w	51.733 (−5.625, 109.092)	47.460 (−9.176, 104.096)	5.144 (−49.395, 59.684)
*p* for trend	0.194	<0.001	<0.001
2back reaction time			
≥6 t/w	0.000	0.000	0.000
2–5 t/w	10.806 (−42.905, 64.517)	5.597 (−45.933, 57.127)	−19.034 (−66.163, 28.095)
≤1 t/w	122.251 (56.380, 188.123)^c^	102.951 (39.060, 166.843)^b^	47.944 (−10.775, 106.662)
*p* for trend	<0.001	<0.001	<0.001
Switching reaction time			
≥6 t/w	0.000	0.000	0.000
2–5 t/w	55.635 (18.451, 92.820)	56.360 (20.246, 92.473)^b^	26.823 (−3.25, 56.896)
≤1 t/w	98.694 (53.091, 144.297)^c^	100.135 (55.359, 144.912)^c^	43.765 (6.297, 81.234)^a^
*p* for trend	<0.001	<0.001	<0.001
Size parity reaction time			
≥6 t/w	0.000	0.000	0.000
2–5 t/w	15.364 (−1.832, 32.559)	14.557 (−2.440, 31.554)	2.097 (−12.820, 17.014)
≤1 t/w	13.708 (−7.380, 34.797)	10.641 (−10.434, 31.716)	−12.720 (−31.306, 5.865)
*p* for trend	0.199	<0.001	<0.001
Conversion function reaction time			
≥6 t/w	0.000	0.000	0.000
2–5 t/w	40.271 (8.008, 72.535)^a^	41.803 (10.200, 73.406)^a^	24.726 (−4.709, 54.161)
≤1 t/w	84.986 (45.417, 124.554)^c^	89.494 (50.311, 128.678)^c^	56.485 (19.812, 93.159)^b^
*p* for trend	<0.001	<0.001	<0.001

Model 1 is a crude model. Model 2 controls for age, gender, father’s education, mother’s education, etc. Model 3 controls for waist circumference, daily moderate to high intensity physical activity, and maximal oxygen uptake (VO_2max_) based on model 2. a indicates *p* < 0.05, b indicates *p* < 0.01, and c indicates *p* < 0.001.

**Figure 2:**
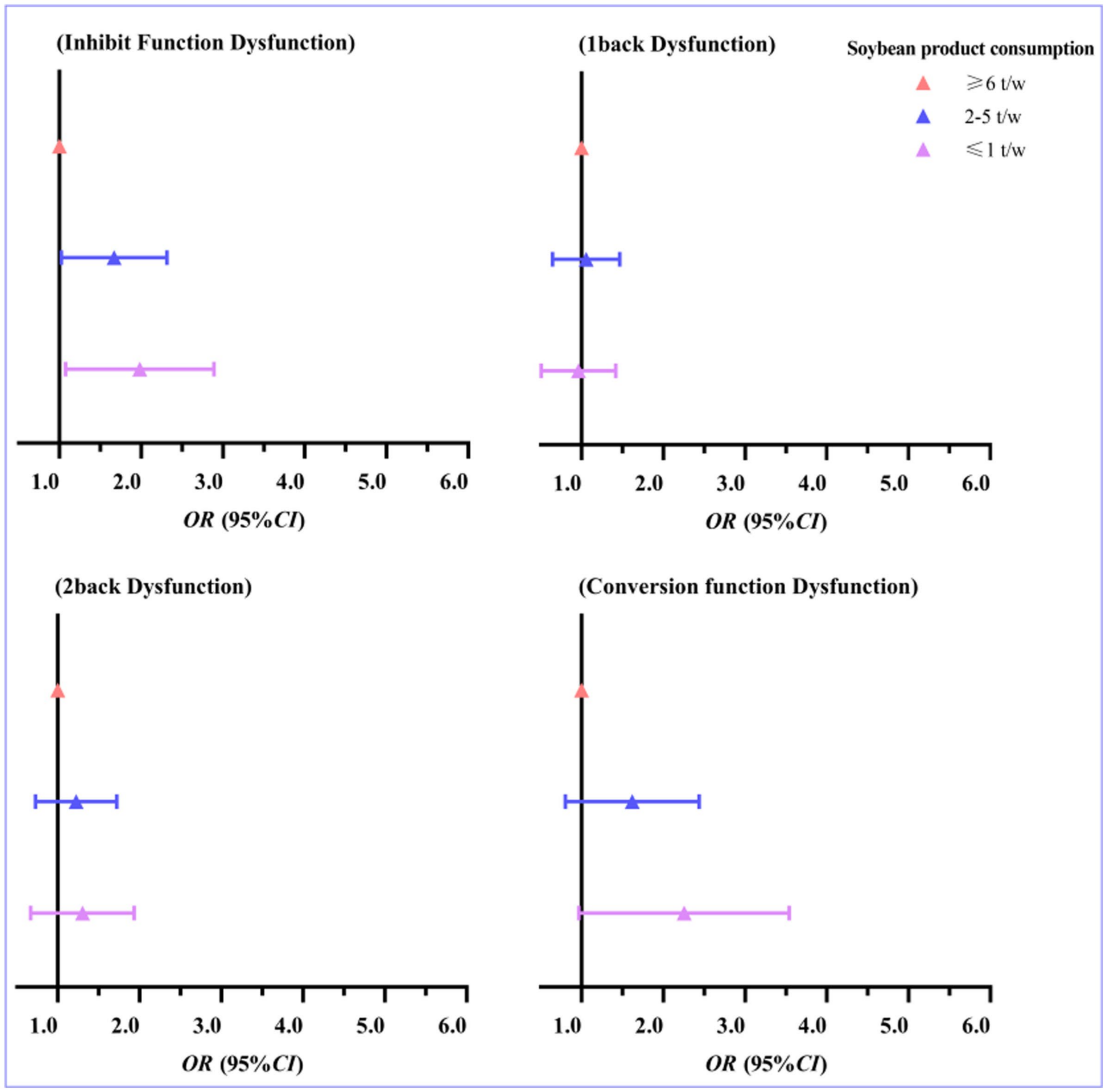
Trends in the ORs of the association between soybean product consumption and executive function.

Binary logistic regression analyses were conducted with the presence of Executive dysfunction as the dependent variable and soybean product consumption as the independent variable among Tibetan children and adolescents in high-altitude areas of Tibet, China. Model 1 was a crude model, model 2 controlled for age, gender, father’s education, mother’s education, etc., and model 3 controlled for waist circumference, daily moderate to high intensity physical activity, and VO_2max_ on the basis of model 2. The results showed that after adjusting for the relevant factors and using the soybean product consumption ≥6 t/w group as the reference group, those with soybean product consumption ≤1 t/w had an increased risk of Inhibit function dysfunction (OR = 1.844, 95% CI: 1.152, 2.951), which was significantly different (*p* < 0.05). In addition, with the soybean product consumption ≥6 t/w group as the reference group, the risk of Conversion function dysfunction was also increased in those with soybean product consumption ≤1 t/w after adjusting for relevant factors (OR = 2.008, 95% CI: 1.106, 3.646), which was significantly different (*p <* 0.05). Overall, the risk of Inhibit function dysfunction and Conversion function Dysfunction increased as soybean product consumption decreased (see Table 4). The trend of the association OR between soybean product consumption and executive function in Tibetan children and adolescents at high altitude in Tibet, China is shown in Figure 2.

**Table 4:** Logistic regression analysis of soybean product consumption and executive function among Tibetan children and adolescents in high altitude areas of Tibet, China.

Executive dysfunction	OR(95% CI)		
	Model 1	Model 2	Model 3
Inhibit function dysfunction			
≥6 t/w	1.000	1.000	1.000
2–5 t/w	1.570 (1.063, 2.319)^a^	1.587 (1.073, 2.347)^a^	1.584 (1.066, 2.353)^a^
≤1 t/w	1.821 (1.155, 2.871)^a^	1.868 (1.177, 2.963)^a^	1.844 (1.152, 2.951)^a^
*p* for trend	<0.001	<0.001	<0.001
1back dysfunction			
≥6 t/w	1.000	1.000	1.000
2–5 t/w	1.146 (0.785, 1.672)	1.146 (0.783, 1.678)	1.002 (0.674, 1.489)
≤1 t/w	1.181 (0.748, 1.865)	1.191 (0.748, 1.896)	0.891 (0.547, 1.450)
*p* for trend	<0.001	<0.001	<0.001
2back dysfunction			
≥6 t/w	1.000	1.000	1.000
2–5 t/w	1.229 (0.828, 1.824)	1.257 (0.845, 1.869)	1.155 (0.762, 1.749)
≤1 t/w	1.507 (0.949, 2.394)	1.633 (1.020, 2.613)^a^	1.198 (0.725, 1.979)
*p* for trend	<0.001	<0.001	<0.001
Conversion function dysfunction			
≥6 t/w	1.000	1.000	1.000
2–5 t/w	1.833 (1.157, 2.906)^a^	1.873 (1.176, 2.981)^a^	1.483 (0.88, 2.498)
≤1 t/w	2.792 (1.675, 4.655)^b^	3.012 (1.787, 5.076)^b^	2.008 (1.106, 3.646)^a^
*p* for trend	<0.001	<0.001	<0.001

Model 1 is a crude model. Model 2 controls for age, gender, father’s education, and mother’s education. Model 3 controls for waist circumference, daily moderate to high intensity physical activity, and maximal oxygen uptake (VO_2max_) on the basis of model 2. a indicates *p* < 0.05, b indicates *p* < 0.01.

## 4 Discussion

The results of the present study showed that the differences in 2back Reaction Time of Tibetan children and adolescents with different soybean product consumption in the high altitude area of Tibet, China, were statistically significant when compared with each other. The differences in Conversion function reaction time were also statistically significant when compared with each other. Overall, it can be seen that with the continuous decline of soybean product consumption, the subjects’ refreshing memory function and conversion flexibility showed a tendency to decrease. Relevant studies have confirmed that soybean product intake in children and adolescents is able to guarantee the necessary protein intake of the body, as well as guaranteeing the continuous supply of energy to the brain, which plays an important role in guaranteeing the function of the brain (36). There are also studies confirming that soybeans and soy products are rich in protein, which is a good source of high-quality protein, and that eating some soy products appropriately every day contributes to the health of the brain and enhances the brain’s memory, which is consistent with the findings of this study (22, 37, 38). In addition, research has confirmed that soybean or soybean products contained in soy lecithin, not only can enhance cellular information transfer capacity, improve brain vitality, but also often called “vascular scavenger,” has to reduce serum cholesterol, reduce blood viscosity, promote blood circulation, prevention of cardiovascular and cerebrovascular diseases and other roles, especially suitable for children and adolescents and the Brain workers (39, 40). Research has also confirmed that soy products are rich in high-quality protein and a variety of amino acids needed by the human body, and these elements help to enhance the function of the cerebrovascular system (41). Soybean fat contains 85.5% of unsaturated fatty acids, including linolenic acid and linoleic acid content, they can reduce cholesterol in the body, the prevention and control of cardiovascular and cerebrovascular diseases and children and adolescents brain health is particularly beneficial (42). In the statement “A Primary Care Agenda for Brain Health” issued by the American Heart Association (AHA), a dietary pattern known as the brain-healthy diet (MIND diet) is recommended to maintain brain health (43). The MIND diet, which combines the DASH diet and the Mediterranean diet, encourages the consumption of nine foods. Nine foods are encouraged and six foods are restricted, with soy products among the encouraged foods recommended as important foods to consume and play an important role in brain health (44).

The results of this study also showed that overall, the risk of Inhibit Function dysfunction and Conversion function dysfunction increased in Tibetan children and adolescents at high altitude as soybean product consumption decreased. This shows that maintaining appropriate soybean product intake has an important role and significance in the development of brain executive function in Tibetan children and adolescents at high altitude. There are also studies confirming that soybeans have a protein content of up to 35%, making them one of the best sources of plant protein. Soybeans and soy products are relatively low in calories, rich in dietary fiber, and contain high levels of unsaturated fatty acids, trace elements, and beneficial phytochemicals such as soy isoflavones, making them an excellent health food (45). Soy products are rich in protein, but also has calcium, magnesium, potassium, soy isoflavones, B vitamins and other nutrients, many benefits to the body, soy protein can make the adult LDL cholesterol reduced by 3 to 4%, this value, although small, but for the prevention of cardio-cerebral vascular disease and the promotion of brain health play a more significant role. Research also confirms (46–48) that soy foods have long been recognized as a source of high-quality protein and healthy fats, such as reducing the risk of coronary heart disease, breast cancer and prostate cancer. It has also been confirmed that the consumption of soy-based foods will have a positive effect on the prevention of neurodegeneration in the brain, thereby effectively contributing to the development of executive functions (49). Yet despite the many benefits of soy, the presence of isoflavones has led to concerns that soy may have adverse effects in some people, concerns that are largely based on animal studies, while human studies support the safety and benefits of soy foods, suggesting a beneficial effect on human health. Of course, the results of studies addressing soy products or soy isoflavones on executive function are not consistent. An experimental study in animals confirmed that repeated daily use of isoflavone metabolites did not alter learning and memory processes in middle-aged rats, with no significant effects (50). In addition, a 6-month experimental study confirmed that 80 mg of soy isoflavone supplementation did not improve performance on standardized neuropsychological tests and overall quality of life in Chinese postmenopausal women (51).

### 4.1 Strengths and limitations

This study has certain strengths and limitations. In terms of strengths, first, this study analyzed for the first time the association between soybean product consumption and executive function in Tibetan children and adolescents at high altitude in China, which provides a reference for the healthy brain development of Tibetan children and adolescents at high altitude. Secondly, the present study adopted the internationally recognized classical executive function paradigm to test inhibitory function (Flanker experimental paradigm), refreshing function (N-back experimental paradigm), and switching function (More-odd Shifting experimental paradigm), and the test results were objective and accurate, which truly reflected the status of the children and adolescents’ executive function. However, this study also has some limitations. First, the sample size of this study is limited and its representativeness is somewhat restricted. Secondly, the covariates included in this study were limited, and future studies should include more relevant covariates, such as covariates for body mass index, economic status, and micronutrients affecting executive function. Third, this study is a cross-sectional study, which can only understand the correlation relationship but not the causal relationship. In the future, a dyadic study should be conducted to understand the association between soybean product consumption and executive function.

## 5 Conclusion

The results of the present study confirm that there is an association between soybean product consumption and inhibitory control and translational flexibility of brain executive functions in Tibetan children and adolescents at high altitude in China, and that decreased soybean product consumption is associated with an increased risk of developing inhibitory control and translational flexibility dysfunctions. Given the results of this study, it is recommended that effective interventions should be implemented in the future to improve soybean product intake among Tibetan children and adolescents to promote healthy brain function. Meanwhile, this study also provides reference and lessons for future brain health interventions for Tibetan children and adolescents in high altitude areas.

## Data availability statement

The raw data supporting the conclusions of this article will be made available by the authors, without undue reservation.

## Ethics statement

The studies involving humans were approved by Human Ethics Committee of East China Normal University (No.: HR0782020). The studies were conducted in accordance with the local legislation and institutional requirements. Written informed consent for participation was not required from the participants or the participants' legal guardians/next of kin in accordance with the national legislation and institutional requirements.

## Author contributions

XY: Conceptualization, Data curation, Methodology, Writing – original draft. FZ: Data curation, Methodology, Writing – review & editing. CB: Data curation, Methodology, Writing – review & editing. YL: Conceptualization, Data curation, Formal analysis, Methodology, Writing – original draft. YG: Data curation, Formal analysis, Writing – review & editing. PS: Formal analysis, Writing – review & editing. JH: Formal analysis, Writing – review & editing.
